# From berries to blocks: carbon stock quantification of a California vineyard

**DOI:** 10.1186/s13021-017-0071-3

**Published:** 2017-02-16

**Authors:** Jorge Andres Morandé, Christine M. Stockert, Garrett C. Liles, John N. Williams, David R. Smart, Joshua H. Viers

**Affiliations:** 1 Environmental Systems, University of California, Merced, Merced, CA USA; 2 Department of Viticulture and Enology, University of California, Davis, Davis, CA USA; 3 College of Agriculture, California State University, Chico, CA USA; 4 Instituto Politécnico Nacional, CIIDIR-Unidad Oaxaca, Santa Cruz Xoxocotlán, Oaxaca Mexico; 5 School of Engineering, University of California, Merced, Merced, CA USA

**Keywords:** Vineyard, Winegrape, Grapevine carbon partitioning, Carbon accounting, Carbon stocks, Aboveground biomass, Allometrics, Carbon sequestration, California

## Abstract

**Background:**

Quantifying terrestrial carbon (C) stocks in vineyards represents an important opportunity for estimating C sequestration in perennial cropping systems. Considering 7.2 M ha are dedicated to winegrape production globally, the potential for annual C capture and storage in this crop is of interest to mitigate greenhouse gas emissions. In this study, we used destructive sampling to measure C stocks in the woody biomass of 15-year-old Cabernet Sauvignon vines from a vineyard in California’s northern San Joaquin Valley. We characterize C stocks in terms of allometric variation between biomass fractions of roots, aboveground wood, canes, leaves and fruits, and then test correlations between easy-to-measure variables such as trunk diameter, pruning weights and harvest weight to vine biomass fractions. Carbon stocks at the vineyard block scale were validated from biomass mounds generated during vineyard removal.

**Results:**

Total vine C was estimated at 12.3 Mg C ha^−1^, of which 8.9 Mg C ha^−1^ came from perennial vine biomass. Annual biomass was estimated at 1.7 Mg C ha^−1^ from leaves and canes and 1.7 Mg C ha^−1^ from fruit. Strong, positive correlations were found between the diameter of the trunk and overall woody C stocks (R^2^ = 0.85), pruning weights and leaf and fruit C stocks (R^2^ = 0.93), and between fruit weight and annual C stocks (R^2^ = 0.96).

**Conclusions:**

Vineyard C partitioning obtained in this study provides detailed C storage estimations in order to understand the spatial and temporal distribution of winegrape C. Allometric equations based on simple and practical biomass and biometric measurements could enable winegrape growers to more easily estimate existing and future C stocks by scaling up from berries and vines to vineyard blocks.

## Background

Agriculture is a key human activity in terms of food production, economic importance and impact on the global carbon cycle. As the human population heads toward 9 billion or beyond by 2050, there is an acute need to balance agricultural output with its impact on the environment, especially in terms of greenhouse gas (GHG) production [1]. An evolving set of tools, approaches and metrics are being employed under the term “climate smart agriculture” (CSA) to help—from small and industrial scale growers to local and national policy setters—develop techniques at all levels and find solutions that strike that production-environment balance and promote various ecosystem services [2, 3]. California epitomizes the agriculture-climate challenge, as well as its opportunities. As the United States’ largest agricultural producing state (2012 farmgate production valued at $44.7 billion, or 11% of the US total) agriculture also accounted for approximately 8% of California’s greenhouse gas (GHG) emissions statewide for the period 2000–2013 [4, 5].

At the same time, California is at the forefront of innovative approaches to CSA [e.g., 6, 7]. Given the state’s Mediterranean climate, part of an integrated CSA strategy will likely include perennial crops, such as winegrapes, that have a high market value and store C long term in woody biomass [8]. Economically, wine production and retail represents an important contribution to California’s economy, generating $61.5 billion in annual economic impact [9]. In terms of land use, 230,000 ha in California are managed for wine production, with 4.2 million tons of winegrapes harvested annually with an approximate $3.2 billion farm gate value [9]. This high level of production has come with some environmental costs, however, with degradation of native habitats, impacts to wildlife, and over abstraction of water resources [see 10].

Although many economic and environmental impacts of wine production systems are actively being quantified, and while there is increasing scientific interest in the carbon footprint of vineyard management activities [e.g., 11], efforts to quantify C capture and storage in annual and perennial biomass remain less well-examined [12, 13]. Studies from Mediterranean climates have focused mostly on C cycle processes in annual agroecosystems or natural systems [14, 15]. Related studies have investigated sources of GHGs [16, 17], on-site energy balance [18], water use [19] and potential impacts of climate change on productivity and the distribution of grape production [20].

The perennial nature and extent of vineyard agroecosystems have brought increasing interest from growers and the public sector to reduce the GHG footprint associated with wine production. The ongoing development of carbon accounting protocols within the international wine industry reflects the increased attention that industry and consumers are putting on GHG emissions and offsets. In principle, an easy-to-use, wine industry specific, GHG protocol would measure the carbon footprints of winery and vineyard operations of all sizes [21]. However, such footprint assessment protocols remain poorly parameterized, especially those requiring time-consuming empirical methods [22]. Data collected from the field, such as vine biomass, cover crop biomass, and soil carbon storage capacity are difficult to obtain and remain sparse, and thus limit the further development of carbon accounting in the wine sector [23]. Simple yet accurate methods are needed to allow vineyard managers to measure C stocks in situ and thereby better parameterize carbon accounting protocols. Not only would removing this data bottleneck encourage broader participation in such activities, it would also provide a reliable means to reward climate smart agriculture.

### Empirical carbon estimation

Building on research that has used empirical data to compare soil and aboveground C stocks in vineyards and adjacent oak woodlands in California [12], this study sought to estimate the C composition of a vine, including the relative contributions of its component parts (root, trunk and cordons, canes, leaves and fruit). By identifying the allometric relationships among trunk diameter, plant height, and other vine dimensions, growers could utilize a reliable mechanism for translating vine architecture and biomass into C estimates [24].

In both natural and agricultural ecosystems, several studies have been performed using allometric equations in order to estimate aboveground biomass to assess potential for C sequestration. For example, functional relationships between the ground-measured Lorey’s height (basal area weighted height of all trees >10 cm in diameter) and aboveground biomass were derived from allometric equations in forests throughout the tropics [25]. Similarly, functional relationships have been found in tropical agriculture for aboveground, belowground, and field margin biomass and C [26–30]. In the vineyard setting, however, horticultural intervention and annual pruning constrain the size and shape of vines making existing allometric relationships less meaningful, though it is likely that simple physical measurements could readily estimate aboveground biomass.

To date, most studies on C sequestration in vineyards have been focused on soil C as sinks [e.g., 13] and some attempts to quantify biomass C stocks have been carried out in both agricultural and natural systems. In vineyards, studies in California in the late 1990s have reported net primary productivity (NPP) or total biomass values between 550 g C m^−2^ (5.5 Mg C ha^−1^) and 1100 g C m^−2^ (11 Mg C ha^−1^) [31]. In terms of spatial distribution, some data of standing biomass collected by Kroodsma et al. [8] from companies that remove trees and vines in California (Noni Enterprises and Orchard Removal, Fresno, California, USA; Wilson Agriculture Company, Shafter, California, USA; Volks and Sons Orchard Removal, Fresno, California, USA) yielded values of 1.0–1.3 Mg C ha^−1^ year^−1^ woody C for nuts and stone fruit species, and 0.2–0.4 Mg C ha^−1^ year^−1^ for vineyards. It has been reported that mature California orchard crops allocate, on average, one third of their NPP to the harvested portion [32] and mature vines 35–50% of the current year’s production to grape clusters [33]. Pruning weight has also been quantified by two direct measurements which estimated 2.5 Mg of pruned biomass per ha for both almonds [34] and vineyards [31].

The incorporation of trees or shrubs in agroforestry systems can increase the amount of carbon sequestered compared to a monoculture field of crop plants or pasture [35]. Additional forest planting would be needed to offset current net annual loss of aboveground C, representing an opportunity for viticulture to incorporate the surrounding woodlands into the system. A study assessing C storage in California vineyards found that on average, surrounding forested wildlands had 12 times more aboveground woody C than vineyards and even the largest vines had only about one-fourth of the woody biomass per ha of the adjacent wooded wildlands [12].

### Study objectives

The objectives of this study were to: (1) measure standing vine biomass and calculate C stocks in Cabernet Sauvignon vines by field sampling the major biomass fractions (i.e., roots, wood, canes, leaves and fruit); (2) calculate C fractions in berry clusters to assess C mass that could be returned to the vineyard from the winery in the form of rachis and pomace; (3) determine proportion of perennially sequestered and annually produced C stocks using easy to measure physical vine properties (i.e., trunk diameter, pruning weights or fruit weights); and (4) develop allometric relationships to provide growers and land managers with a method to rapidly assess vineyard C stocks. Lastly, we validate block level estimates of C with volumetric measurements of vine biomass generated during vineyard removal.

## Methods

### Study site

**Fig. 1:**
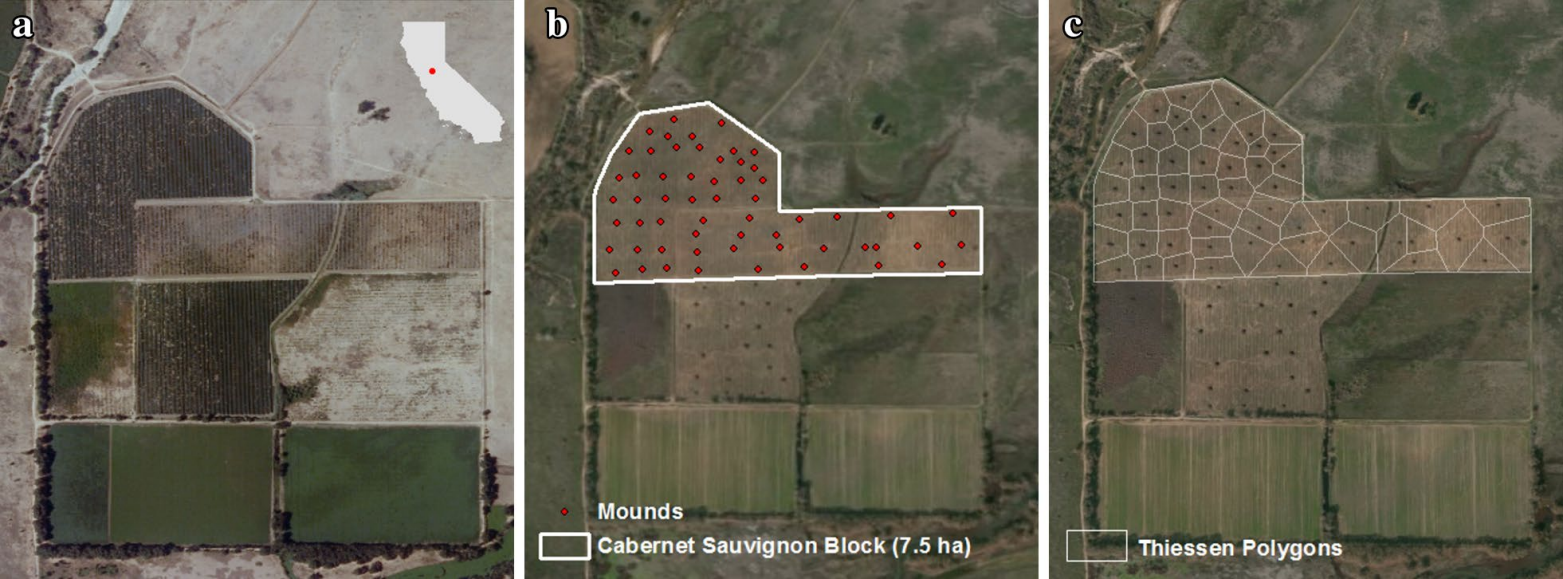
Study vineyard in southern Sacramento County, California, USA. The vineyard prior to removal is shown in **a**. The boundaries of the Cabernet Sauvignon plot are outlined in **b** with *dots* representing woody debris mounds resulting from uprooted vines. The Thiessen polygons around mounds in **c** were used to estimate source area for C per hectare estimates

The study site is located in southern Sacramento County, California, USA (121°22′33″W, 38°18′19″N; Fig. 1a), and the vineyard is part of a property annexed into a seasonal floodplain restoration program, which has since removed the levee preventing seasonal flooding. The ensuing vineyard removal allowed destructive sampling for biomass measurements and subsequent C quantification.

The vineyard is considered part of the Cosumnes River appellation within the Lodi American Viticultural Area, a region characterized by its Mediterranean climate—cool wet winters and warm dry summers—and by nearby Sacramento-San Joaquin Delta breezes that moderate peak summer temperatures compared to areas north and south of this location. The study site is characterized by a mean summer maximum air temperature of 32 °C, has an annual average precipitation of 90 mm, typically all received as rain from November to April [36]. During summer time, the daily high air temperatures average 24 °C, and daily lows average 10 °C. Winter temperatures range from an average low 5 °C to average high 15 °C [37]. Total heating degree days for the site are approximately 3420 [38] and the frost-free season is approximately 360 days annually [24].

Similar to other vineyards in the Lodi region, the site is situated on an extensive alluvial terrace landform formed by Sierra Nevada outwash with a San Joaquin Series soil (fine, mixed, active, thermic Abruptic Durixeralfs). This soil-landform relationship is extensive, covering approximately 160,000 ha across the eastern Central Valley and it is used extensively for winegrape production. The dominant soil texture is clay loam with some sandy clay loam sectors; mean soil C content, based on three characteristic grab samples processed by the UC Davis Analytical Lab, in the upper 8 cm was 1.35% (sd = 0.77%) and in the lower 8–15 cm was 1.1% (sd = 0.1%).

**Fig. 2:**
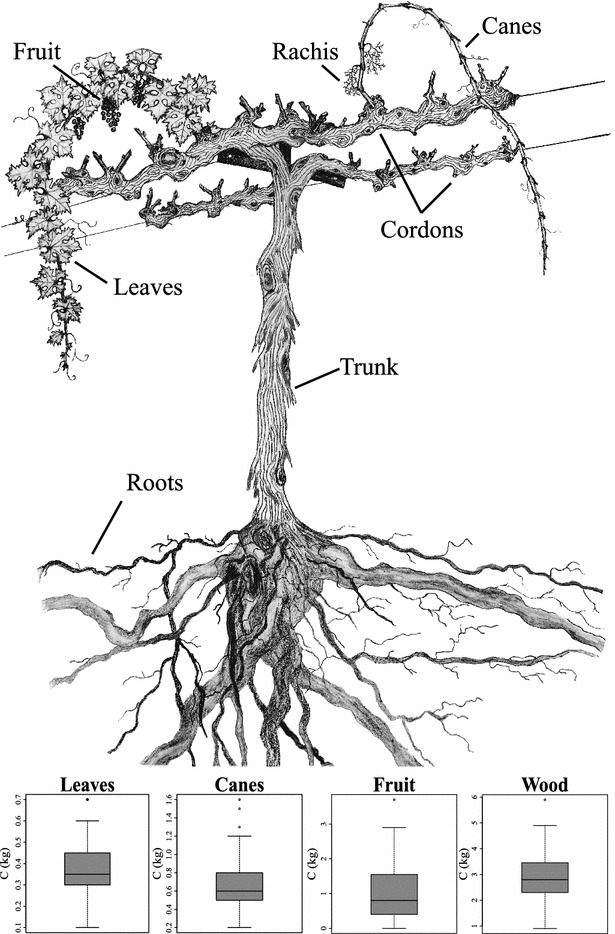
Vine diagram plus *boxplots* describing major categories of C stocks measurements. Fruit was weighed separately in berries, seeds and rachis. Cordons represent the *horizontal arms* where the canes grow from. *Boxplots* show the median and range of C stocks for four categories in kg C vine^−1^. Histogram-*boxplot* (*bottom*) shows the total C distribution per vine (kg C plant^−1^) yielded by 72 samples (artwork credit C. M. Stockert)

The vineyard plot consisted of 7.5 ha of Cabernet Sauvignon vines, planted in 1996 at a density of 1631 plants ha^−1^ (3.35 m by 1.83 m spacing) with flood irrigation during spring and summer seasons. The vines were trained using a quadrilateral trellis system with two parallel cordons and a modified Double Geneva Curtain structure attached to T-posts (Fig. 2). Atypically, these vines were not grafted to rootstock, which is used often in the region to modify vigor or limit disease (i.e., phylloxera).

### Standing biomass quantification

In Sept.–Oct. of 2011, aboveground biomass was measured from 72 vines. The vineyard (7.5 ha) was divided equally in twelve randomly assigned blocks, and six individual vines from each block were processed into major biomass categories of leaf, fruit, cane and trunk plus cordon (Fig. 2). Grape berry clusters were collected in buckets, with fruit separated and weighed fresh in the field. Leaves and canes were collected separately in burlap sacks, and the trunks and cordons were tagged. Biomass was transported off site to partially air dry on wire racks and then fully dried in large ventilated ovens.

Plant tissues (i.e., leaves, canes, wood, roots, grape skins, rachis and seeds) were dried at 60 °C for 48 h and then ground to pass through a 250 μm mesh sieve using a Thomas Wiley^®^ Mini-Mill (Thomas Scientific, Swedesboro, NJ). Total C (%) in plant tissues was analyzed using a PDZ Europa ANCA-GSL elemental analyzer at the UC Davis Stable Isotope Facility. For cluster and berry C estimations, grape clusters were randomly selected from all repetitions. Berries were removed from cluster rachis. While the berries were frozen, the seeds and skins were separated from the fruit flesh or “pulp”, and combined with the juice (denoted juice + pulp herein). The rachis, skins and seeds were dried in oven and weighed. The pulp was separated from the juice + pulp with vacuum filtration using a pre-weighed Q2 filter paper (1–5 μm retention, Fisher Scientific). The filter paper with pulp was oven dried and weighed to get insoluble solid fraction (pulp). The largest portion of grape juice soluble solids are sugars. Sugars were measured at 25% using a Refractometer PAL-1 (Atago USA, Inc., Bellevue, WA). The C content of sugar was calculated at 42% using the formula of sucrose.

Belowground biomass was measured by pneumatically excavating the root system with compressed air applied at 0.7 Mpa (100 psi) for three of the 12 sampling blocks, exposing two vines each in 8 m^3^ pits. The soil was pre-wetted prior to excavation to facilitate removal and minimize root damage. A root restricting duripan, common in this soil, provided an effective rooting depth of about 40 cm at this site with only 5–10 fine and small roots (generally <20 mm diameter) able to penetrate below this depth in each plot. Roots were washed, cut into smaller segments and separated into four size classes (<2, 2–6, 6–20 and >20 mm), oven-dried at 60 °C for 48 h and weighed. Larger roots were left in the oven for 4 days. Stumps (i.e., fraction of the plant immediately above the roots but belowground) were considered part of the root system for this analysis.

### C estimates

In vineyard ecosystems, annual C is represented by fruit, leaves and canes, and is either removed from the system and/or incorporated into the soil C pools, which was not considered further. Structures whose tissues remain in the plant (i.e., trunk plus cordons and roots) were considered perennial C. Woody biomass volumes were measured and used for perennial C estimates. Cordon and trunk diameters were measured using a digital caliper at four locations per piece and averaged, and lengths were measured with a calibrated tape. Sixty vines were used for the analysis; twelve vines were omitted due to missing values in one or more vine fractions. All statistical estimates were conducted in *R* [39].

### Mound volume and mass estimation

An earthmoving machine was used to uproot vines and gather them together to form mounds. Twenty-six mounds consisting of trunks plus cordons and canes were measured across this vineyard block (Fig. 1b). The mounds represented comparable spatial footprints within the vineyard area (Fig. 1c). Mound C stocks were estimated using their biomass contribution areas, physical size, density and either a semi-ovoid or hemispherical model.

#### Physical size

A real-time kinematic (RTK) global positioning system (Topcon HiperV) was used to map boundaries of each mound, with vertices placed every 1.5 m to measure circumference. Average mound height (m) was calculated using a stadia rod and laser inclinometer range finder. The circumsurficial distance (distance between two points measured across the pile surface) over the major axes (N–S and E–W) of each mound was measured with a calibrated cord. Combined, these measurements were used to estimate pile volume using semi-ovoid and hemispherical models (see below).

#### Semi-ovoid model

For the semi-ovoid model, length [*l*] and width [*w*] and mound height *(h)* were determined from direct field measurements. The mound volume was calculated as:

$$V_{m} = \frac{2}{3}\pi \cdot l \cdot w \cdot h$$

#### Hemispheric model

The hemispheric model used mapping data to resolve the geometric centroid in ArcGIS (v10 ESRI, Redlands) from each perimeter. A circular area from the average radius of each mound centroid to perimeter vertex was then regressed against mapped mound area, resulting in an area-adjusted model. This area was back-transformed to arrive at best-fit radius [*h*] for each mound to represent one-half the volume of a sphere:

$$V_{m} = \frac{2}{3}\pi h^{3}$$

#### Mound C density

A standardized volume (~0.085 m^3^) of biomass was collected by cutting out random sections of the same area from 12 mounds using a plastic container to insure size consistency. Plant material in the mounds included the fractions of trunk plus cordons, roots and canes, and the way the mound elements fill out the container simulated their spatial arrangement in the mound. Samples represent a range of biomass configurations (relative ratio of biomass volume:void) found across the vineyard block. Sample contents were divided into vine biomass classes (canes, wood, and roots) dried, and weighed. Relating sample mass with the collection volume supports the calculation of mound density (47.5 kg/m^3^) and C mass. Vine category proportion data were compared to the measured vine proportion data to validate the basic assumption supporting these calculations. All biomass data were multiplied by a factor of 0.47 (average C calculated for the three fractions) to estimate C mass (kg). C data were scaled up to the individual mound, unit area, and vineyard totals (Mg ha^−1^).

## Results

### Vine C stocks

**Fig. 3:**
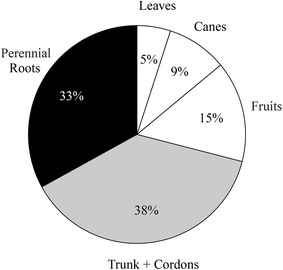
Average percentage distribution of fractions of biomass. C (kg plant^−1^) based on 72 aboveground and 12 belowground samples of 15 year old Cabernet Sauvignon grapevines. *White slices* represent annual biomass C, and *black* and *grey slices* indicate perennial biomass C

Average C stocks per vine partition out to roughly equal one-third fractions of (i) roots, (ii) trunk plus cordons, and (iii) leaves, fruit and canes (Table 1; Fig. 3). The vine-based values when scaled to the spatial extent of the vineyard give an estimate of 12.3 Mg C ha^−1^ across the 7.5 ha site (92.3 Mg C total) based on 1631 vines ha^−1^ density. When partitioned into annual versus perennial contributions, 3.4 Mg C ha^−1^ (28%) was found to come from annual production in canes, leaves and fruit, and the remaining 8.9 C ha^−1^ (72%) was stored in the perennial woody fraction (trunk plus cordons and roots) (Table 2; Fig. 2). Boxplots in Fig. 2 show the variability of total C by biomass category.

**Table 1:** Average biomass, and C for vine fractions and total plant are shown

Biomass fractions	n	% C	Dry biomass		C stocks		C stocks	
			kg vine^−1^		kg C vine^−1^		Mg C ha^−1^	
Fruit	60	43	2.6	(2.0)	1.1	(0.9)	1.7	(1.4)
Leaves	60	45	0.9	(0.3)	0.4	(0.2)	0.6	(0.3)
Canes	60	48	1.4	(0.6)	0.7	(0.3)	1.1	(0.5)
Wood	60	48	6.2	(2.1)	3.0	(1.0)	4.8	(1.6)
Roots	3	44	5.7	(0.9)	2.5	(0.4)	4.1	(0.6)
Total			16.8	(4.4)	7.7	(2.0)	12.3	(2.5)

Standard deviations (sd) are shown in parentheses

**Table 2:** Fractions of C sequestration in vines by time and space

C distribution	Biomass fraction	Mg C ha^−1^	Total	%
Temporal				
Annual	Fruit	1.7	3.4	28
	Leaves	0.6		
	Canes	1.1		
Perennial	Wood	4.8	8.9	72
	Roots	4.1		
Spatial				
Aboveground	Fruit	1.7	8.2	67
	Leaves	0.6		
	Canes	1.1		
	Wood	4.8		
Belowground	Roots	4.1	4.1	33

Annual growth represents the seasonal vegetative and reproductive development starting in spring and finishing in early fall. Most fruit is removed whereas leaves and canes return to soil. Wood constitutes the sum of trunk plus cordons

**Fig. 4:**
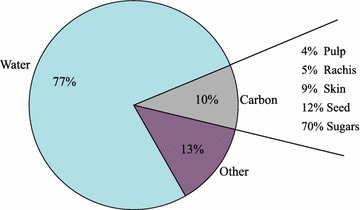
Grape cluster C. The total mass of a fresh grape cluster showing the proportion of C. The C fraction is then partitioned into the components of a berry. Pulp is defined as the fruit flesh

Total C stocks per plant were variable (sd = 2.0 kg/26%). Fruit harvest accounted for approximately 10% of vine C by weight, about 26% of this C could be turned into soil C storage by returning the rachis and pomace (skin and seed) to the soil (Fig. 4), a source of biomass C contributing to long term C storage in agroecosystems and beneficial for GHG mitigation purposes. In this vineyard, the amount of return would average 0.44 Mg C ha^−1^ or 13% of the annual C fraction.

Per vine C was calculated to be 46% (7.7 kg; sd = 2.0 kg) of total dry biomass per vine (average = 16.8 kg), resulting in approximately one-third of each vine in annual (29%), aboveground wood (38%), and belowground root wood (33%) C (Fig. 3). Root C content was estimated as 44% of dry weight, of which 83.7% was stored in roots >6 mm diameter (including stump), and only ~4% was found in fine roots <2 mm diameter (Table 3).

**Table 3:** Biomass and C fractions for five different root diameter classes including the stump

Root diameter classes (mm)	Root Biomass (Mg ha^−1^)	Root C (Mg C ha^−1^)	Fraction (%)
<2	0.4	0.16	3.8
2–6	1.2	0.51	12.5
6–20	2.9	1.27	31.3
>20	2.6	1.16	28.6
Stump	2.2	0.97	23.8
Total	9.3	4.1	100

Estimations per hectare are based on vine spacing = 1.83 × 3.35 (1631 vines ha^−1^)

**Fig. 5:**
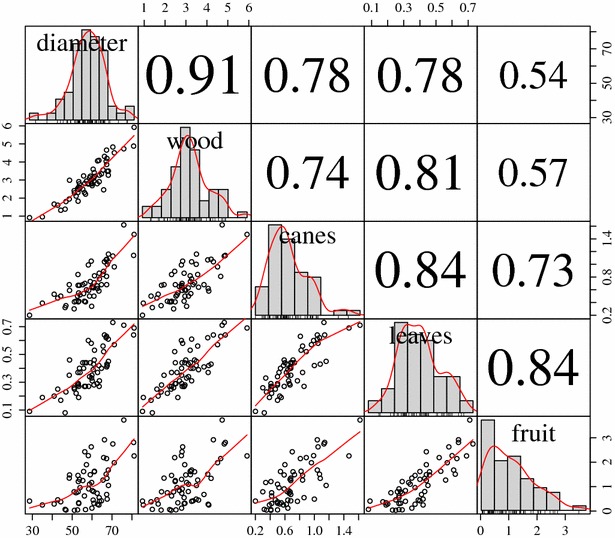
*Scatterplot* matrix showing correlations between vine components. Vine trunk diameter (mm) and vine C stocks (kg C vine^−1^) fractions in wood (trunk plus cordons, i.e. permanent scaffold), canes, leaves and fruit (grape clusters including rachis) are shown. The *lower left triangle* below the diagonal shows *scatterplots* with loess curves (*x* and *y axis* indicating values for each variable). The frequency histograms in the diagonal include kernel density estimation curves (only *x axis* indicating values for the variable). The *upper right triangle* shows Pearson correlation coefficients with all levels of significance p < 0.001, (n = 60)

**Fig. 6:**
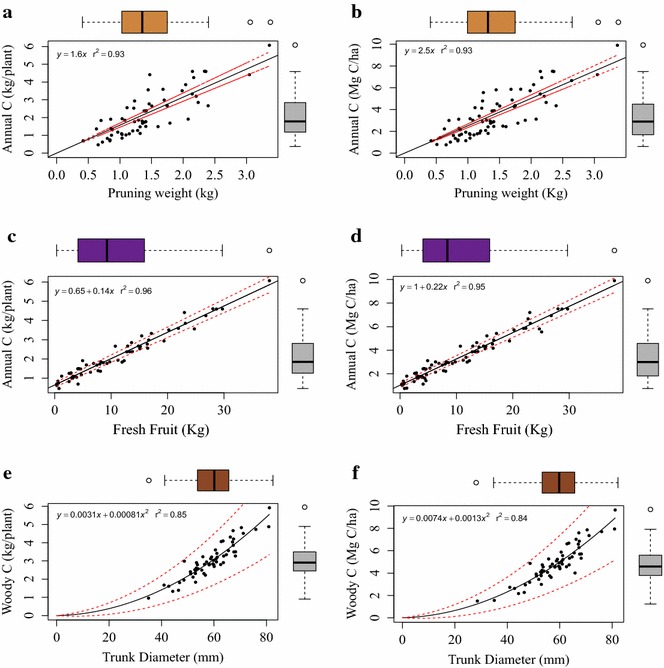
Linear and quadratic vine allometrics with 95% confidence intervals. Allometrics for (**a**) pruning weight and annual C stocks (*R* ^*2*^ = 0.93, p < 0.001), **b** fresh fruit weight and annual C stocks (*R* ^*2*^ = 0.96, p < 0.001), and **c** trunk diameter and woody C stocks (*R* ^*2*^ = 0.85, p < 0.001) are shown. Annual C stocks represent the C content of canes, leaves and fruit together. Woody C stocks represent the C content of trunk plus cordons (perennial wood). Allometrics scaled to block level response (Mg C ha^−1^) at planting density are also shown for pruning weight (**d**), wet fruit (**e**), and trunk diameter (**f**)

Carbon stock estimates in woody tissue correlated positively with trunk diameter (r = 0.91) and more strongly than with other biomass categories of canes (r = 0.74), leaves (0.81), and fruit (r = 0.57) (Fig. 5). All three of the allometric relationships developed here—wet fruit weight, trunk diameter and pruning weight—showed relatively robust coefficients of determination when regressed against C content (Fig. 6) (i.e., R^2^ > 0.85).

### Mound C stocks

The secondary approach to estimate C stocks by fitting the regular hemispherical and semi-ovoid models produced comparable average biomass values. Our estimations of C stocks per ha quantifying mound biomass yielded an average of 9.93 ± 2.7 (semi-ovoid model) and 10.57 ± 3.6 Mg C ha^−1^ (hemispherical model) (Table 4). This compares favorably to 10.02 ± 1.9 Mg C ha^−1^ obtained by standing biomass considering C stocks estimations of trunk plus cordons, roots and canes (Table 1). Additionally, a paired *T* test was run to compare differences between the two mound methods finding no significant differences (95% CI; p = 0.2). A Welch Two Sample t test applied to check for possible significant differences between the Standing biomass and mound methods found no significant difference (95% CI p = 0.72).

**Table 4:** Physical comparisons of biomass C measurements in vineyard block

Method	Model	Number of samples	Mean (Mg C ha^−1^)	Range (Mg C ha^−1^)	Sd (σ)
Standing biomass		60	10.02	5.9–16.2	1.9
Mounds	Semi-ovoid	26	9.93	6.2–17.0	2.7
	Hemispherical		10.57	5.9–23.4	3.6

Mounds included trunk plus cordons and canes. Estimations for standing biomass consider only the elements included in mounds to make them comparable

## Discussion

The present study provides results for an assessment of vineyard biomass that is comparable with data from previous studies, as well as estimates of belowground biomass that are more precise than previous reports. While most studies on C sequestration in vineyards have focused on soil C, some have quantified aboveground biomass and C stocks. For example, a study of grapevines in California found net primary productivity (NPP) values between 5.5 and 11 Mg C ha^−1^ [31]—figures that are comparable to our mean estimate of 12.4 Mg C ha^−1^. For pruned biomass, our estimate of 1.1 Mg C ha^−1^ (2.3 Mg biomass ha^−1^) were comparable to two assessments that estimated 2.5 Mg of pruned biomass ha^−1^ for both almonds [34] and vineyards [31]. Researchers reported that mature orchard crops in California allocated, on average, one third of their NPP to harvestable biomass [32], and mature vines allocated 35–50% of that year’s production to grape clusters [33]. Our estimate of 50% of annual biomass C allocated to harvested clusters represent the fraction of the structures grown during the season (1.7 out of 3.4 Mg C ha^−1^). Furthermore, if woody annual increments were considered (i.e., estimating differences between two seasons by applying our “trunk diameter” model) this proportion would be even lower. Likewise the observed 1.7 Mg ha^−1^ in fruit represents ~14% of total biomass (1.7 out of 12.3 Mg C ha^−1^), which is within 10% of other studies in the region at similar vine densities [i.e., 40]. More importantly, this study reports the fraction of C that could be recovered from winemaking and returned to the soil for potential long term storage. However, this study is restricted to the agronomic and environmental conditions of the site, and the methodology would require validation and potential adjustment in other locations and conditions.

Few studies have conducted a thorough evaluation of belowground vine biomass in vineyards, although Elderfield [41] did estimate that fine roots contributed 20–30% of total NPP and that C was responsible for 45% of that dry matter. More recently, Brunori et al. [13] studied the capability of grapevines to efficiently store C throughout the growing season and found that root systems contributed to between 9 and 26% of the total vine C fixation in a model *Vitis vinifera sativa* L. cv Merlot/berlandieri rupestris vineyard.

The results of our study provide a utilitarian analysis of C storage in mature wine grape vines, including above- and belowground fractions and annual vs. perennial allocations. Such information constitutes the basic unit of measurement from which one can then estimate the contribution of wine grapes to C budgets at multiple scales—fruit, plant or vineyard level—and by region, sector, or in mixed crop analyses.

Our study builds on earlier research that focused on the basic physiology, development and allocation of biomass in vines [33, 42, 43]. Previous research has also examined vineyard-level carbon at the landscape level with coarser estimates of the absolute C storage capacity of vines of different ages, as well as the relative contribution of vines and woody biomass in natural vegetation in mixed vineyard-wildland landscapes [12]. The combination of findings from those studies, together with the more precise and complete (i.e. detailed, measurement-based above- and below-ground C estimates) carbon-by-vine structure assessment provided here, mean that managers now have access to methods and analytical tools that allow precise and detailed C estimates from the individual vine to whole-farm scales.

As carbon accounting (including offsets, credits and payments) in vineyard landscapes becomes more sophisticated, widespread and economically relevant, such vineyard-level analyses will become increasingly important for informing management decisions. The greater vine-level measuring precision that this study affords should also translate into improved scaled-up C assessments (e.g., county-, state- or sector-wide). In California alone, for example, there are more than 230,000 ha are planted in vines [21]. Given that for many, if not most of those hectares, the exact number of individual vines is known, it is easy to see how improvements in vine-level measuring accuracy can have benefits from the individual farmer to the entire sector.

Previous efforts to develop rough allometric woody biomass equations for vines notwithstanding [12], there is still a need to improve our precision in estimating of how biomass changes with different parameters. Because the present analysis was conducted for 15 year old Cabernet vines, there is now a need for calibrating how vine C varies with age, varietal and training system. There is also uncertainty around the influence of grafting onto rootstock on C accumulation in vines. As mentioned in the methods, the vines in this study were not grafted—an artifact of the root-limiting duripan approximately 50 cm below the soil surface. The site’s location on the flat, valley bottom of a river floodplain also means that its topography, while typical of other vineyard sites per se, created conditions that limit soil depth, drainage and decomposition. As such, the physical conditions examined here may differ significantly from more hilly regions in California, such as Sonoma and Mendocino counties. Similarly, the lack of a surrounding natural vegetation buffer at this site compared to other vineyards (e.g., [12]) may mean that the ecological conditions of the soil communities may or may not have been broadly typical of those found in other vineyard sites. Thus, to the extent that future studies can document the degree to which such parameters influence C accumulation in vines or across sites, they will improve the accuracy and utility of C estimation methods and enable viticulturists to be among the first sectors in agriculture for which accurate C accounting is an industry-wide possibility.

The current study was also designed to complement a growing body of research focusing on soil-vine interactions [44–46]. Woody carbon reserves and sugar accumulation play a supportive role in grape quality, the main determinant of crop value in wine grapes. The extent to which biomass production, especially in belowground reservoirs, relates to soil carbon is of immediate interest for those focused on nutrient cycling, plant health and fruit production, as well as for those concerned with C storage [44, 47, 48].

The soil-vine interface may also be the area where management techniques can have the highest impact on C stocks and harvest potential [45, 46, 49, 50]. We expect the belowground estimates of root biomass and C provided here will be helpful in this regard and for developing a more thorough understanding of belowground C stores at the landscape level. For example, Williams et al. [12] estimated this component to be the largest reservoir of C in the vineyard landscape they examined, but they did not include root biomass in their calculations. Others have assumed root systems to be ~30% of vine biomass based on the reported biomass values for roots, trunk, and cordons [40]. With the contribution of this study, the magnitude of the belowground reservoir can now be updated.

## Conclusions

Wine is a commodity of worldwide importance, and vineyards constitute a significant land use and contribution to economies across Mediterranean biome and beyond [10, 51]. Like orchards and tree plantations, grapevines are a perennial crop that stores C long-term in woody tissue, thereby helping to mitigate GHG emissions. Our study provides estimates of C in grape vines by vine component, as well as a simple measurement tool kit that growers can use to estimate the C in their vines and vineyard blocks. The equations presented here represent some of the first allometric models for estimating grapevine C from berries to blocks, with the hope that widespread use and refinement of these techniques may lead to recognition and credit for the C storage potential of vineyards and other perennial woody crops, such as orchards. The successful implementation of these methods, if applied widely to multiple cropping systems, could improve the precision of measurement and the understanding of C in agricultural systems relative to other human activities.

## References

[1] Godfray HCJ, Beddington JR, Crute IR, Haddad L, Lawrence D, Muir JF (2010). Food security: the challenge of feeding 9 billion people. Science.

[2] Lipper L, Thornton P, Campbell BM, Baedeker T, Braimoh A, Bwalya M (2014). Climate-smart agriculture for food security. Nat Clim Change.

[3] Palm C, Blanco-Canqui H, DeClerck F, Gatere L, Grace P (2014). Conservation agriculture and ecosystem services: an overview. Agric Ecosyst Environ.

[4] California environmental protection agency air resources board. California GHG Emission Inventory 2015. http://www.arb.ca.gov/cc/inventory/pubs/reports/ghg_inventory_trends_00-13%20_10sep2015.pdf. Accessed 18 Sep 2016.

[5] United States Department of Agriculture. California Agricultural Statistics, 2012 Crop Year: National Agricultural Statistics Service, Pacific Regional Office; 2013. www.nass.usda.gov/Statistics_by_State/California/Publications/California_Ag_Statistics/Reports/2012cas-all.pdf. Accessed 15 Sep 2016.

[6] Haden VR, Dempsey M, Wheeler S, Salas W, Jackson LE (2013). Use of local greenhouse gas inventories to prioritise opportunities for climate action planning and voluntary mitigation by agricultural stakeholders in California. J Environ Plan Manag.

[7] Jackson L, Haden VR, Wheeler SM, Hollander AD, Perlman J, O’Geen T, et al. Vulnerability and adaptation to climate change in California agriculture. California Energy Commission; 2012.

[8] Kroodsma DA, Field CB (2006). Carbon sequestration in California Agriculture, 1980–2000. Ecol Appl.

[9] Wine Institute G-F. Gomberg-Fredrikson Report. California Dept. of Food & Agriculture, US Tax & Trade Bureau, and US Dept. of Commerce; 2009.

[10] Viers JH, Williams JN, Nicholas KA, Barbosa O, Kotze I, Spence L (2013). Vinecology: pairing wine with nature. Conserv Lett.

[11] Marras S, Masia S, Duce P, Spano D, Sirca C (2015). Carbon footprint assessment on a mature vineyard. Agric For Meteorol.

[12] Williams J, Hollander A, O’Geen A, Thrupp L, Hanifin R, Steenwerth K (2011). Assessment of carbon in woody plants and soil across a vineyard-woodland landscape. Carbon Balance Manage.

[13] Brunori E, Farina R, Biasi R (2016). Sustainable viticulture: the carbon-sink function of the vineyard agro-ecosystem. Agric Ecosyst Environ.

[14] Andrews SS, Mitchell JP, Mancinelli R, Karlen DL, Hartz TK, Horwath WR (2002). On-farm assessment of soil quality in California’s central valley. Agron J.

[15] Veenstra JJ, Horwath WR, Mitchell JP (2007). Tillage and cover cropping effects on aggregate-protected carbon in cotton and tomato. Soil Sci Soc Am J.

[16] Carlisle E, Smart DR, Williams LE, Summers M. California vineyard greenhouse gas emissions: Assessment of the available literature and determination of research needs. California Sustainable Winegrowing Alliance; 2010.

[17] Alsina MM, Fanton-Borges AC, Smart DR (2013). Spatiotemporal variation of event related N2O and CH4 emissions during fertigation in a California almond orchard. Ecosphere.

[18] Kavargiris SE, Mamolos AP, Tsatsarelis CA, Nikolaidou AE, Kalburtji KL (2009). Energy resources’ utilization in organic and conventional vineyards: energy flow, greenhouse gas emissions and biofuel production. Biomass Bioenergy.

[19] Herath I, Green S, Singh R, Horne D, van der Zijpp S, Clothier B (2013). Water footprinting of agricultural products: a hydrological assessment for the water footprint of New Zealand’s wines. J Clean Prod.

[20] Hannah L, Roehrdanz PR, Ikegami M, Shepard AV, Shaw MR, Tabor G (2013). Climate change, wine, and conservation. Proc Natl Acad Sci.

[21] Wine Institute and International Partners to Release New Greenhouse Gas Protocol and Accounting Tool [press release]. San Francisco; 2008.

[22] Forsyth K, Oemcke D. International Wine Carbon Calculator Protocol Version 1.2. Provisor Pty Ltd and Yalumba Wines, Hartley Grove, Urrbrae, SA 5064. Australia; 2008. p. 152.

[23] Schultz HR (2010). Climate change and viticulture: research needs for facing the future. J Wine Res.

[24] Chave J, Andalo C, Brown S, Cairns M, Chambers J, Eamus D (2005). Tree allometry and improved estimation of carbon stocks and balance in tropical forests. Oecologia.

[25] Saatchi SS, Harris NL, Brown S, Lefsky M, Mitchard ETA, Salas W (2011). Benchmark map of forest carbon stocks in tropical regions across three continents. Proc Natl Acad Sci.

[26] D’Acunto L, Semmartin M, Ghersa CM (2014). Uncropped field margins to mitigate soil carbon losses in agricultural landscapes. Agric Ecosyst Environ.

[27] Henry M, Tittonell P, Manlay R, Bernoux M, Albrecht A, Vanlauwe B (2009). Biodiversity, carbon stocks and sequestration potential in aboveground biomass in smallholder farming systems of western Kenya. Agric Ecosyst Environ.

[28] Kuyah S, Dietz J, Muthuri C, Jamnadass R, Mwangi P, Coe R (2012). Allometric equations for estimating biomass in agricultural landscapes: I. Aboveground biomass. Agric Ecosyst Environ.

[29] Kuyah S, Dietz J, Muthuri C, Jamnadass R, Mwangi P, Coe R (2012). Allometric equations for estimating biomass in agricultural landscapes: II. Belowground biomass. Agric Ecosyst Environ.

[30] Tadesse G, Zavaleta E, Shennan C (2014). Effects of land-use changes on woody species distribution and above-ground carbon storage of forest-coffee systems. Agric Ecosyst Environ.

[31] Christensen LP. Raisin production manual: UCANR Publications; 2000.

[32] Rufat J, DeJong TM (2001). Estimating seasonal nitrogen dynamics in peach trees in response to nitrogen availability. Tree Physiol.

[33] Williams LE, Christensen LP (2000). Growth and development of grapevines. Raisin production manual.

[34] Holtz B, McKenry M, Caesar-TonThat T (2004). Wood chipping almond brush and its effect on the almond rhizosphere, soil aggregation and soil nutrients. Acta Hortic.

[35] Sharrow S, Ismail S (2004). Carbon and nitrogen storage in agroforests, tree plantations, and pastures in western Oregon, USA. Agrofor Syst.

[36] Kleinschmidt Associates. Cosumnes River preserve management plan—final. Report. Kleinschmidt Associates; 2008.

[37] NOAA. Technical Memorandum NWS WR-272: Climate of Sacramento, CA. Technical memorandum. National Oceanic and Atmospheric Administration—Department of Commerce, USA; 2005. Contract No.: WR-272.

[38] BizEE Software Unlimited. Custom Degree Day Data- Cosumnes River, Wilton, CA, US (121.23 W, 38.44 N) 2016. http://www.degreedays.net/. Accessed 12 Sept 2016.

[39] R Core Team. R: a language and environment for statistical computing. In: R Foundation for Statistical Computing V, Austria. Vienna; 2015.

[40] Keightley KE, Bawden GW (2010). 3D volumetric modeling of grapevine biomass using Tripod LiDAR. Comput Electron Agric.

[41] Elderfield H, Schlesinger WH. Biogeochemistry. An analysis of global change, xiii + 588 pp. San Diego, London, Boston, New York, Sydney, Tokyo, Toronto: Academic Press. Price US $49.95 (paperback). ISBN 0 12 625155 X. CHAMEIDES, WL & PERDUE, EM 1997. Biogeochemical Cycles. A Computer-Interactive Study of Earth System Science and Global Change. xi + 224 pp. + disk. New York, Oxford: Oxford University Press. Price£ 37.50 (hard covers). ISBN 0 19 509279 1. Geological Magazine. 1998;135(06):819–42.

[42] Williams LE, Zamski E, Schaffer AA (1996). Grape. Photoassimilate distribution in plants and crops: source–sink relationships.

[43] Williams LE, Biscay PJ (1991). Partitioning of dry weight, nitrogen, and potassium in Cabernet Sauvignon grapevines from anthesis until harvest. Am J Enol Vitic.

[44] Eldon J, Gershenson A (2015). Effects of cultivation and alternative vineyard management practices on soil carbon storage in diverse Mediterranean landscapes: a review of the literature. Agroecol Sustain Food Syst.

[45] Simansky V (2013). Soil organic matter in water-stable aggregates under different soil management practices in a productive vineyard. Arch Agron Soil Sci.

[46] Steenwerth K, Belina KM (2008). Cover crops enhance soil organic matter, carbon dynamics and microbiological function in a vineyard agroecosystem. Appl Soil Ecol.

[47] Suddick EC, Ngugi MK, Paustian K, Six J (2013). Monitoring soil carbon will prepare growers for a carbon trading system. Calif Agric.

[48] Zarraonaindia I, Owens SM, Weisenhorn P, West K, Hampton-Marcell J, Lax S (2015). The soil microbiome influences grapevine-associated microbiota. Mbio.

[49] Bosco S, Di Bene C, Galli M, Remorini D, Massai R, Bonari E (2013). Soil organic matter accounting in the carbon footprint analysis of the wine chain. Int J Life Cycle Assess.

[50] Agnelli A, Bol R, Trumbore SE, Dixon L, Cocco S, Corti G (2014). Carbon and nitrogen in soil and vine roots in harrowed and grass-covered vineyards. Agric Ecosyst Environ.

[51] Underwood EC, Viers JH, Klausmeyer KR, Cox RL, Shaw MR (2009). Threats and biodiversity in the mediterranean biome. Divers Distrib.

